# How Do Adolescents Use Social Networks and What Are Their Potential Dangers? A Qualitative Study of Gender Differences

**DOI:** 10.3390/ijerph19095691

**Published:** 2022-05-07

**Authors:** Giulio de Felice, Jessica Burrai, Emanuela Mari, Fabrizio Paloni, Giulia Lausi, Anna Maria Giannini, Alessandro Quaglieri

**Affiliations:** 1 Faculty of Literature and Philosophy, Sapienza University of Rome, 00185 Rome, Italy; giulio.defelice@uniroma1.it; 2 Xenophon College London, University of Chichester, London PO19 6PE, UK; 3 Department of Psychology, Sapienza University of Rome, 00185 Rome, Italy; jessica.burrai@uniroma1.it (J.B.); fabrizio.paloni@uniroma1.it (F.P.); giulia.lausi@uniroma1.it (G.L.); annamaria.giannini@uniroma1.it (A.M.G.); alessandro.quaglieri@uniroma1.it (A.Q.)

**Keywords:** social networks, adolescents, Grounded-Theory, haters, violence, gender differences

## Abstract

The rapid development of software applications and the increasing use of the Internet have raised many questions about the impact of this technology on the lives of adolescents, especially on “digital natives.” The advent of social networks (SNs) restructures their relationships in various ways, affecting both adolescents’ development and mental health. The present study aims to investigate uses and dangers of SNs according to a sample of 296 (166 female and 130 male) Italian middle and high schools adolescents (age range 13–18) and build a model of how SNs can turn out to be dangerous. To achieve this, twenty-four audio-recorded focus groups of Italian male and female adolescents were investigated by a Grounded Theory approach, abstracting from the transcripts the main uses and dangers of SNs and proposing a final model for the interpretation of the whole set of categories. The results highlighted two main dangers of SNs: (a) the desperate search for popularity, and (b) the exhibition of violent or offensive behavior facilitated by the sense of protection and anonymity derived from being hidden behind a virtual account. Finally, a psychological model of how SNs can turn out to be dangerous is presented. This study could be useful in developing prevention procedures against the risks of SNs (e.g., cyberbullying, internet addiction) without demonizing the use of social media as such.

## 1. Introduction

Mobile phone technology and the Internet have become an integral part of the daily interactions and activities of individuals, especially adolescents [1,2]. This generation, often referred to as “digital natives,” has been exposed to technology from birth [3]. The idea that digital devices and the Internet have a lasting influence on the way humans develop, socialize, and thrive seems to have great relevance [4,5], especially as the time spent online by young people has doubled in the last decade [6]. The rapid development of software applications and the increasing use of the Internet raise many questions about the impact of this technology on the lives of adolescents [7]. Subrahmanyam et al. [8] hypothesized that adolescents bring into social media the problems of their “offline” lives with regard to identity construction, peer group relations, sexuality, sensation seeking, and risk taking, aspects typically faced during this period of psychophysical development [9].

Recent studies [10,11] have indicated that social media use among 13–17-year-olds is around 93–97%, and since the introduction of social media, such as Facebook and Instagram, the scientific community has questioned whether such massive use affects an adolescent’s wellbeing and health. However, results to date have been dissenting [12,13,14,15,16].

According to Strasbuger [17] and his “Super peer” theory, social media, unlike face-to-face interactions, seems to exert a strong influence and pressure on adolescents, forcing them toward risky behaviors that are, instead, represented as normative. A further theory, the “Facebook Influence Model” [18], describes social media, in contrast to face-to-face situations or “traditional” media, as a mechanism for amplifying peer influence in which a behavioral reinforcement is manifested in the form of “likes” and/or comments as well as in the possibility of interacting directly or indirectly with a wider network of people outside one’s peer group.

One aspect that should not be underestimated, closely linked to the use of SNs and their ability to “cross borders,” is related to online violence. We refer here to “hate speech” (or “cyberhate”), a form of online aggression toward individuals based on their race, gender, nationality, sexual orientation, ethnicity, religion, or disability with the aim of promoting hostility, discrimination, and/or violence [19]. Hate speech can manifest itself through highly offensive online posts, comments, messages, videos, or images [20,21]. The difference between hate speech and cyberbullying, cyber-harassment, or cyberstalking is that these latter forms are typically directed against an individual or a small group of individuals [22], while hate speech is directed toward specific social subgroups or a group of people representative of that given subgroup [23,24].

Several studies have shown that adolescent online experiences with cyberhate are relatively common. Cyberhate can be offensive, cruel, or threatening, and can be expressed through degrading online writing or speech, such as posts, comments, text messages, videos, or images [20,21]. Violent and discriminatory behavior performed “online” by young people generates a rush of excitement related to the aspects of recognition and approval and, additionally, the negative consequences of the actions committed often go unpunished, thus favoring a process of de-responsibilization [25]. Although there are no direct consequences associated with violent actions online, there are harmful consequences in the use of SNs including health problems [26,27], emotional problems, Internet addiction [28,29,30], and self-harm, including suicide [31]. The SNs may also provide benefits, such as a perceived greater connection with others when, for instance, in-person social interactions are severely limited (e.g., the COVID-19 pandemic; [32,33]. In addition, positive relationships among peers [34], family, neighborhood, and school apparently mitigate some negative outcomes such as delinquency and risky behavior [35].

Thus, the potentials and risks of SNs for adolescent development and mental health should still be clarified. For example, it is unclear whether the number of accounts owned, or the frequency with which they are checked, can affect psychosocial functioning [36].

There are gender differences in the use of the Web, particularly in the use of SNs. In fact, several studies have shown that interaction with SNs is greater with females than males. In particular, girls spend more time on social media, smartphones, and computers for social networking [37], while boys are more likely to use the Web for online gaming [37,38,39,40,41]. Furthermore, males can be more heavily influenced by marketing strategies and are at higher risk of developing behavioral addiction [42].

By contrast, girls are more likely to use social media as a platform for social comparison and feedback on their appearance and personal value [43,44]. Arguably, this attitude could be the result of girls becoming more self-objectified (i.e., placing more emphasis on how their physical bodies appear to others; [45]. Indeed, the use of SNs is linked to specific concerns about body weight, especially among adolescent girls [46]. The relationship between time spent on SNs and symptoms of anxiety and depression is stronger for girls than boys [47]. This is consistent with other contributions indicating that girls may be more sensitive than boys to feedback given to them on SNs, thus, reacting with higher levels of distress [48,49].

### Study Aim

The present study can be included in the above-mentioned scientific literature. Its main aim is to study the uses and potential dangers of SNs among a sample of Italian adolescents.

In particular:

RQ1: What specific characteristics of use and dangerousness do SNs have?

RQ2: Are there any differences in the use and dangerousness of SNs among boys versus girls?

RQ3: Is it possible to create a model that could explain how SNs can turn out to be dangerous and the mechanism of use and negative reinforcement in the use of SNs?

## 2. Materials and Methods

### 2.1. Study Design

This paper is based on Grounded Theory: a qualitative approach to the analysis of interviews or focus groups ideal for abstracting the main categories in which they are organized [50]. Its purpose is to generate a model that explains the variability of the data rather than testing an a priori hypothesis. This approach is mainly based on three steps: *open coding*, *axial coding*, and *selective coding*. Researchers must constantly review the connections between abstract categories and data until the latter are completely saturated. In other words, the research team’s work ends when the abstract model explains the greater amount of data variance. At this point the new complete theoretical framework has been developed [51,52]. 

### 2.2. Ethical Issues

All study procedures were carried out in accordance with the Declaration of Helsinki. The Institutional Review Board of the Department of Psychology, Sapienza University of Rome (protocol number 1450/2021) approved the procedures and the accompanying consent forms.

### 2.3. Participants

The dataset we analyzed consists of twenty-four focus groups carried out and recorded within Italian middle and high schools (age range 13–18). The focus groups were carried out in 6 middle schools and 6 high schools, respectively. Each class (consisting of about 24 students) was divided in half in order to conduct 24 focus groups.

Each focus group was led by a psychologist experienced in leading groups of adolescents and young adults and included approximately twelve participants. The total number of participants was 296, comprising 166 girls and 130 boys.

### 2.4. Procedural Section

Data were collected by using twenty-four focus groups in twelve schools (i.e., Italian middle and high schools), consisting of open-ended questions covering violence and aggression in SNs (for more detailed information see Section 2.4.1). 

All the focus groups were conducted at the beginning of the pre-pandemic period, from September to December 2019.

Participants were interviewed in their classrooms, and each interview lasted about 60 to 90 min. The interviews were conducted without the presence of the teacher, with the aim of making students feel freer to express their opinions.

The collection of data was concluded when a saturation point was reached and new interviews did not seem to make any substantial contribution to the model previously generated on early data. The interviews were taped and transcribed verbatim and analyzed according to the Grounded Theory. Data were examined line by line in order to identify students’ opinions, feelings, and actions related to the themes mentioned in the interviews. Codes maintained words used by the scholars in order to maintain the semantic of the data, to verify their descriptive content, and to confirm that they were “grounded” in the data. 

#### 2.4.1. Focus Group Questions

The core theme of the focus groups was violence and aggression in SNs. The focus groups were conducted using thirteen open questions and leaving the participants’ discussion free until no one had anything more to add. The open questions were the following: 

1. How do you communicate with your friends, classmates, and family members when you are not physically together?

2. What digital communication tools do you use? (e.g., WhatsApp, Instagram, Facebook, Snapchat, Twitter, WeChat, Viber, TikTok, others?)

3. Which ones do you enjoy the most? Why?

4. How much time do you spend daily on electronic devices (e.g., Smartphone, tablet, computer, play station, X-box)? For what kind of activities, mainly?

5. Have you ever come into direct contact with content that you consider inappropriate and/or violent? Targeting you personally or others? What was it about? What were your reactions? 

6. Is it different for you to communicate aggressively online versus face to face? What do you think is different?

7. In your opinion, are there specific categories of people who can be particularly vulnerable to this inappropriate behavior? (Do gender-related issues come up? If they don’t come up spontaneously, ask question 7a)

7a. “There are many statistics indicating that on social networks there is a prevalence of insulting communications, with words and/or images, directed towards women and girls. Do you have any feedback or experience in relation to that?”

8. Are there behaviors and/or attitudes that when displayed on SNs are more likely to lead to being attacked? Which ones? Why?

9. Who, in your opinion, most frequently communicates aggressively on SNs? What effect does this have on the targeted people, groups, and specific social categories?

10. What are the reasons for this aggressive behavior on SNs?

11. The following is a case that actually happened: a boy created a closed group on Facebook where he posted photos of some of his friends (girls) taken from social media. He then invited other friends (boys) to make sexually explicit comments about the girls. Participants enthusiastically commented, but at a certain point one of the girls discovered what happened. How do you think the girl reacted?

12. Do you know how to recognize an aggression against a person, a group, or a social category on SNs? How do you notice it?

13. In your opinion, can these online aggressions have consequences? What kind of consequences?

### 2.5. Data Analysis

The transcripts of the focus groups were analyzed, highlighting the similarities and differences in the responses of male and female adolescents. In particular, 75% of the transcripts were analyzed, leaving 25% for the theoretical saturation test. The first step of analysis, *open coding*, is based on dividing the entire transcript into analyzable text segments. Through continuous comparison and brainstorming, the categories that can best represent the meaning of a specific segment of text are abstracted [53]. The second step of Grounded Theory is called *axial coding*. In this further step, the categories that emerged during the open coding phase are reanalyzed and the relationships between them are highlighted. The group of researchers, therefore, abstracts second-level categories which can group the categories that emerged from the open coding process. The last step in Grounded Theory is *selective coding*. The goal of selective coding is to connect together, in a single model, the relationships between the second-level categories that emerged during the axial coding phase. This process culminates in showing the reader a theoretical framework suitable to explain most of the variability of the data. The data (the first-level categories and the second-level categories) are finally reanalyzed making sure that new possible categories would not improve the accuracy of the proposed model. Finally, the theoretical saturation test was performed: an analysis of the remaining 25% of the transcript using the first and second level categories that emerged.

## 3. Results

In the open coding phase, after multiple reviews, the group of researchers extracted 10 categories. Examples of the process of forming these categories are shown in Table 1.

In the axial coding phase, the group of researchers extracted two main second-level categories that contain the categories of the open coding phase of analysis. These are: (a) Uses of SNs and (b) Dangers of SNs. Table 2 below shows the contents of each category.

**Figure 1:**
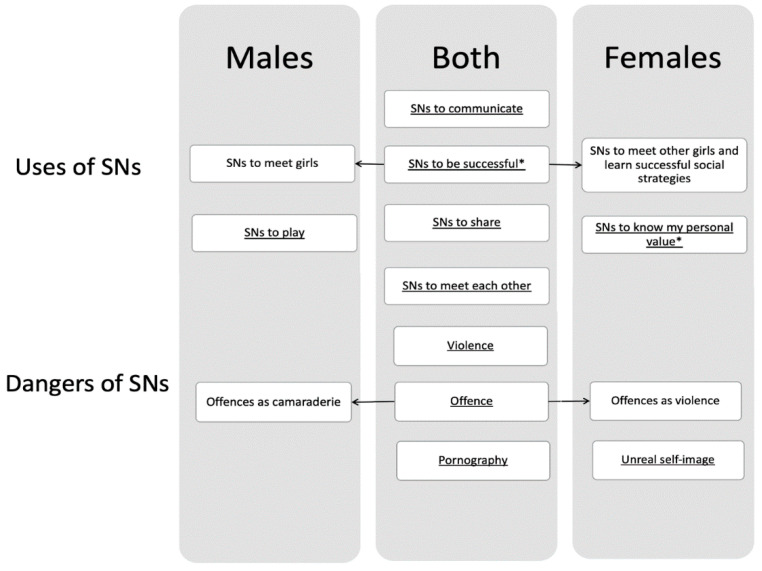
Relations between the first and second-level categories and the gender of the participants. Note. Items with * represent the riskiest uses of SNs, exposing the subject to potential dangers.

Included in the “Uses of SNs” we highlight “the use of SNs to know my personal value” and “the use of SNs to be successful” as the riskiest attitudes towards SNs, exposing the subject to potential dangers. During the selective coding phase, a complete theoretical framework was proposed to explain most of the data variability. The following figure shows the relationships existing between the first and second-level categories, highlighting similarities and differences between male and female adolescents (Figure 1).

**Figure 2:**
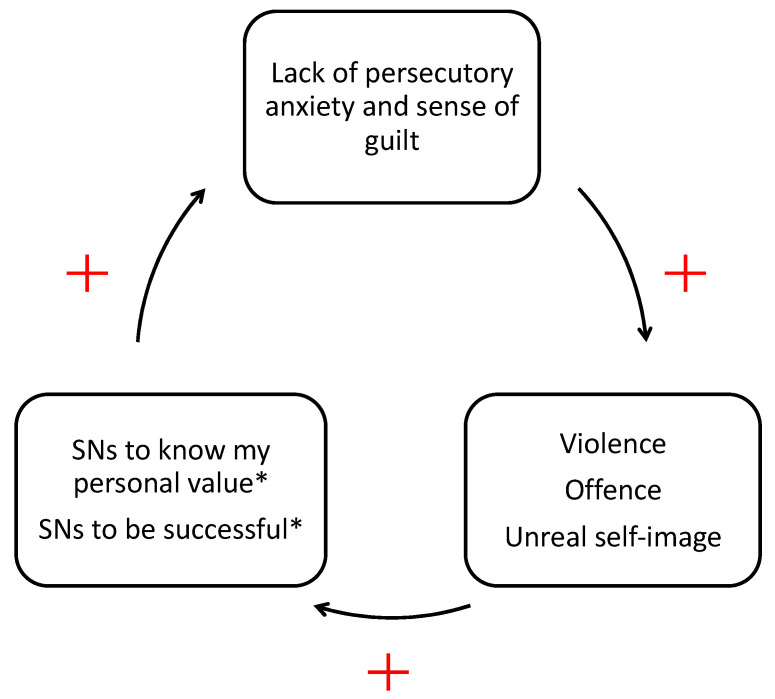
A psychological model on how SNs can turn out to be dangerous. Note. Items with * represent the riskiest uses of SNs, exposing the subject to potential dangers.

In Figure 1, we have included the categories common to males and females in the center, with the categories belonging to males on the left and the categories belonging to females on the right. Two common categories (“SNs to meet each other” and “Offence”) convey a different meaning depending on whether it is a male or a female adolescent who is speaking. These differences were shown through arrows. The theoretical saturation test applied to the remaining 25% of the data did not generate any further possible category and showed the accuracy of the proposed classification in describing the entire dataset. Finally, a psychological model on how SNs can turn out to be dangerous tools is proposed. We highlighted the interactions among the riskiest uses of SNs and how they mutually reinforce each other (Figure 2).

## 4. Discussion

The present study aimed to investigate uses and dangers of SNs according to a sample of Italian adolescents and build a model for how SNs can turn out to be dangerous. The first two research questions were: (1) what specific characteristics of use and dangerousness do SNs have? (RQ1); and (2) are there any differences in the use and dangerousness of SNs among boys versus girls? (RQ2).

As we can evince from Figure 1, the majority of the categories belong to both male and female adolescents. In fact, both genders use social networks to communicate with peers (“SNs to communicate”) and to share images or information (“SNs to share”). Both genders also use SNs to meet each other and meet new people (“SNs to meet each other”), but while for males this means meeting new girls for a possible relationship, for girls it is intended as meeting other girls to support each other. Furthermore, it is mainly males who use social networks to play online games (“SNs to play”), while it is mainly females who use social networks to get feedback on their own value (“SNs to know my personal value”). In this case, the personal value is assigned on the basis of likes and comments received—in other words, on the basis of popularity. In addition, both males and females use social networks to try to have money and success (“SNs to be successful”). This sometimes happens by uploading comments or extreme videos aimed at obtaining as many likes as possible. Examples described by the participants concern a boy who wants to show his ability to set fire to a sofa and eventually the fire spreads throughout the whole house, or a girl who films herself trying to throw herself under a train and is seriously injured, or boys who heavily insult people who have many followers in order to get a lot of comments, and finally, a group of boys who record a homeless man having a seizure and upload the image online instead of calling an ambulance. It is this equation (i.e., “popularity” = “personal value”) that we believe to be a potentially very dangerous ingredient for adolescent wellbeing. In fact, regarding the major dangers that adolescents highlight in the use of SNs, we find four categories: “Violence”; verbal “offences” or offences shown through videos and photos; “pornography”; and receiving an “unreal self-image.” Here we must highlight a difference in how male and female adolescents perceive offences committed in SNs. For males, these are often perceived as camaraderie. The atmosphere of SNs for males often mimics that of the team sports locker room in which the teammates offend each other to make the group laugh. For females, on the other hand, the offences are perceived exclusively as violent acts. However, consistent with the existing literature [5,6,7,12], all participants highlight how frequently violent or offensive content is present in SNs. While the existing literature often highlights the problem of cyberbullying, our results also shed light on another phenomenon. In fact, the females in our study emphasize a further potentially dangerous aspect: coming into contact via SNs with an image of an ideal girl which turns out to be very frustrating for them. Here are the words of a participant:

*“I uninstalled social media from my phone, and I was much calmer because I no longer saw the stereotype of a girl I am supposed be every day. Since I took it off, it seems strange to say, but my self-esteem has risen more because I always saw types of girls that I knew I couldn’t be.”*

The constant exposure to an “ideal model” who is perceived to be very distant from their current condition results in a very pronounced feeling of frustration, especially in girls. Furthermore, adolescents point out a final ingredient that characterizes SNs: the lack of persecutory anxiety and sense of guilt. In the words of one participant:

*“Hiding yourself behind an account, perhaps sometimes fake, causes the insults and offences to start very quickly, and the person has no worries about what can happen after this behavior.”*

There are numerous contributions in the literature that underline the importance of the sense of guilt and persecutory anxiety as a key mechanism in the formation of a less sadistic and more adequate-to-reality “Super-Ego” [54,55]. In other words, the lack of persecutory anxiety and sense of guilt generates a violent and offensive vicious circle that is sometimes difficult to break (Figure 2). This leads to the third research question of this study: is it possible to create a model that could explain how SNs can turn out to be dangerous? (RQ3).

Some adolescents sign into SNs eager for recognition of their personal value and therefore are sensitive to the judgments of others. Some people present in SNs often exhibit violent or offensive behavior due to (a) the sense of protection they perceive by being hidden behind a virtual account and (b) the effort directed toward being successful and popular. These two ingredients, taken together, cause the lack of persecutory anxiety and sense of guilt in those groups exhibiting such behaviors. This type of conduct is particularly frustrating for those adolescents needing recognition of their personal value. In fact, these latter can react in a depressive way, feeling sadness and dejection, or in an aggressive way, populating the group of persons who exhibit violent or offensive behaviors, increasingly in search of popularity, increasingly with the perception of being protected behind a virtual account, increasingly with a lack of persecutory anxiety and sense of guilt (Figure 2). To our knowledge, this is the first time that a model has been proposed in the literature for how SNs can become dangerous for adolescents. The existing literature often focuses on cyberbullying (e.g., 7, 12), more rarely on the problems of envy and popularity (e.g., 6), almost never on the relational ingredients of SNs that promote their emergence.

Finally, we emphasize how that vicious circle (Figure 2) is fed by two different types of frustration: frustration coming from violent or offensive behavior, and frustration coming from the constant exposure to an ideal and unattainable self-image (i.e., what in this study we have called “Unreal self-image” and in the literature goes under the name of “Ego-Ideal”). In fact, in the literature it has been highlighted how the indirect frustration coming from the “Ego-Ideal” can be as unbearable as the direct frustration coming from the “Super-Ego” [56,57,58,59,60]. We have called the frustration deriving from the Ego-Ideal “indirect” because it comes from the perceived distance between the ideal self-image and the real self, and we have named the frustration deriving from the Super-Ego “direct” because it represents the introjected moral rules. SNs, therefore, as well as being useful tools in the service of social relations and knowledge, can unfortunately also represent a global, and very frustrating, Ego-Ideal (for the gap between the ideal self and the real self) and/or Super-Ego (for the exhibition of violent and offensive behaviors).

This study, while showing important characteristics of the uses and dangers of SN, is certainly not devoid of limitations. The most important lies in the sample size, which cannot be considered representative of Italy as a whole. However, we intend to replicate the work by enlarging the study sample. Finally, another important limitation lies in the lack of quantitative measures to complement the results obtained by means of a purely qualitative methodology. We believe that a quali-quantitative approach can be used in the replication of the present study.

## 5. Conclusions

This study shows important characteristics of the uses and dangers of social networks. Our findings highlighted two main dangers of SNs (the desperate search for popularity and the exhibiting of violent or offensive behavior) which seem to be facilitated by the sense of protection derived from being hidden behind a virtual account. The effort to be popular and the sense of protection cause a lack of persecutory anxiety or sense of guilt, which allows violent and offensive behaviors to be implemented. In fact, it is much easier to exhibit such behaviors without the fear of being persecuted or hurt by others. To our knowledge, this is the first time that a model has been proposed in the literature for how SNs can become dangerous for adolescents. The model has various clinical implications; among them, we highlight: (a) in the case of a patient who suffered of cyberbullying, the health professional (e.g., general practitioner, psychologist, psychiatrist, or psychotherapist) should pay attention to the patient’s internal fragilities that sometimes facilitate such a violent relational dynamic. Specifically, the use of SNs to increase one’s popularity and to know or confirm one’s personal value can be grounded on scarce self-esteem (i.e., scarce benign narcissism), which should be addressed in order to make the patient less dependent on others’ judgments; (b) in the case of a patient who committed cyberbullying, the health professional should instead pay attention to the suffering embedded in such behavior. Sometimes, with the extreme suffering due to the patient’s needs not being adequately seen, the patient’s thoughts and emotions are systematically discarded and derided. In summary, the violent or offensive act often represents an extreme attempt to seek help, to seek an environment capable of catering to the patient’s care.

Social media is a pervasive part of modern social life and represents an artificial world in which there is a growing desire to present ideal representations of oneself as an extension of offline identity in which users present relatively authentic versions of themselves. This allows users to create a “virtual self” by performing or editing the content that is presented to others. The artificiality of these platforms highlights a constant tendency toward self-idealization that could be harmful to individual wellbeing.

While a growing body of research suggests a mixed effect (i.e., both positive and negative) of social media use on wellbeing [5,61,62,63], our findings, in line with the study by Bailey et al., 2020 [64], suggest that social media can in a sense help individual wellbeing. However, this depends on the way these platforms are used; indeed, while creating an “enhanced” self-figure can be beneficial on the one hand, an authentic self-expression is always preferable and psychologically beneficial on the other. Future research could assess the role of individual differences in self-expression on social media in order to avoid the development of social media addiction. This study aims to highlight the uses and dangers of social networks according to a sample of Italian adolescents for developing prevention procedures against the risks of SNs (e.g., cyberbullying, internet addiction) without demonizing the use of social media as such.

## Figures and Tables

**Table 1:** Examples of the open coding analysis.

Number	Category	Original Transcript
1	SNs to Communicate	I use social media to talk to friends. Sometimes I ask them if they want to play on PlayStation and while we play, we talk.
2	SNs to meet each other	I use social media to meet my friends. I send a message to our group, and everyone sees it. In this way it is much easier to meet them.
3	SNs to play	Sometimes I spend hours and hours playing because there is nothing to do. When I play with my cousins with a special server that gives us the possibility to play all together, I stay connected a lot. If I am alone at home I am bored.
4	SNs to share	We don’t use social media a lot, but they are useful for sharing homework. Sometimes I don’t make them all, there are often a lot of them and after school I’m tired.
5	SNs to know my personal value	In SNs I include my photos and photos with my friends. I wait to see the reactions and if others like me. This also happens with comments. I always wait for the reactions of others to see if they appreciate what I write.
6	SNs to be successful	I remember when I signed up, I wanted to see if I was successful with others, if they liked my posts and photos. I didn’t expect to be popular but just that others liked me. Since I got a girlfriend, I’ve been online a lot less, connected a lot less.
7	Violence	A while ago there was a video of a homeless man at the station who either took drugs or had a seizure. Some guys were filming him on the phone, others were kicking him. I felt bad looking at it. Almost vomiting. There are tons of videos like that.
8	Offence	There are people that I don’t even know who comment on my photos saying that “I suck.” I don’t say anything, I’m sad.
9	Pornography	Well, it happened to me that some older men sent me pictures of their penis. I blocked them immediately and made the report.
10	Unreal self-image	I uninstalled social media from my phone, and I was much calmer because I don’t see the stereotype of a girl I should be every day. Since I took it off, it seems strange to say, but my self-esteem has risen more because I always saw types of girls that I knew I couldn’t be.

**Table 2:** Content of axial coding categories.

Category Number	Open Coding Category	Axial Coding Category
1	SNs to communicate	Use of SNs
2	SNs to meet each other	
3	SNs to play	
4	SNs to share	
5	SNs to know my personal value *	
6	SNs to be successful *	
7	Violence	Dangers of SNs
8	Offence	
9	Pornography	
10	Unreal self-image	

Note. Items with * represent the riskiest uses of social networks, exposing the subject to potential dangers.

## Data Availability

He data presented in this study are available on request from the corresponding author. The data are not publicly available due to privacy considerations.
